# Supplementary material for: Feasibility of Predicting Static Dielectric Constants of Polymer Materials: A Density Functional Theory Method
Source: Polymers (Basel). 2021 Jan 17;13(2):284. doi: 10.3390/polym13020284 (PMC7830834; doi:10.3390/polym13020284)
Supplement: Supplementary file 1 [file polymers-13-00284-s001.pdf]

Supplementary Material:

Feasibility of predicting static dielectric constant of polymer materials: A density functional theory method

Zheng Tang[a], Chaofan Chang[a], Feng Bao[a], Lei Tian[a], Huichao Liu[a], Mingliang Wang[a]* , Caizhen Zhu[a]*, Jian Xu[a]

[a]Institute of Low-dimensional Materials Genome Initiative, College of Chemistry and Environmental Engineering, Shenzhen University, Shenzhen, Guangdong, 518060, People's Republic of China.

S.1 The derivation of the Clausius-Mossotti equation

According to the classical electromagnetic theory, when the external electric field is applied on the dielectric medium, the positive and negative charges in the materials will deviate from their equilibrium positions by very small distances throughout the volume of the materials. Thus, the electric dipoles are produced. The process of producing electric dipoles which are oriented along the field direction is called polarization in the dielectric medium. If we consider polarization at the molecular level, the effect of the applied external electric field is to induce the electric dipole on the individual molecule. For the dielectric medium with *N* molecules per unit volume and each molecule has a dipole moment μ, the total polarization *P* can be given by:

$$P = N\mu \tag{S-1}$$

In addition, the induced dipole moment μ is proportional to the local electric field or internal field E_i:

$$\mu = \alpha E_i \tag{S-2}$$

where α is the polarizability of the individual molecule. Therefore, the total polarization *P* of the dielectric medium containing *N* molecules is given by:

$$P = N\alpha E_i \tag{S-3}$$

According to the approach adopted by Lorentz [1], the local electric field in the dielectric medium is given by:

$$E_i = E + \frac{P}{3\varepsilon_0} \tag{S-4}$$

Here *E* is the magnitude of the applied external electric field. ε_0 is the permittivity of

free space. By substituting equation (3) into equation (4) and simplifying the equation (3), one can get:

$$P = \frac{N\alpha E}{\left(1 - \frac{N\alpha}{3\varepsilon_0}\right)} \tag{S-5}$$

Moreover, according to the classical electromagnetic theory, the total polarization P also can be given by:

$$P = \varepsilon_0 \left(\varepsilon_r - 1\right) E \tag{S-6}$$

Here ε_r is the dielectric constant, also called the relative permittivity of the dielectric medium. Therefore, by combining the equation (5) and (6), then eliminate the external field E, one can get the equation as below:

$$\frac{\varepsilon_r - 1}{\varepsilon_r + 2} = \frac{N\alpha}{3\varepsilon_0} \tag{S-7}$$

This equation is known as the Clausius-Mossotti equation which relates the microscopic property polarizability α of the molecule to the macroscopic property dielectric constant ε_r of the dielectric medium. It is named after two outstanding scientists Ottaviano-Fabrizio Mossotti and Rudolf Clausius.

S.2 The details on the computational setup

During the simulations, two Intel(R) Xeon(R) Platinum P8136 CPUs are employed. The number of cores used in typical calculations is 56. The typical elapsed time of the calculations for the three polymer materials with 6 monomers is outlined in Table.S-1. From Table.S-1, we can see that the most time-consuming jobs are the geometric optimizations. It takes only a few hours to complete all the calculations for single polymer material.

Table.S-1. The elapsed time of the calculations for the three polymer materials. The unit of all the elapsed time is in minutes.

Type	Geometric optimization	Polarizability calculation	Volume calculation
PE	60	5	2
PTFE	90	7	3
PS	269	31	7

S.3 Cartesian coordinates of all stationery points determined

The Cartesian coordinates of all stationary points determined for the three polymer materials with 6 monomers are summarized as follows:

(1) The Cartesian coordinates of PE with 6 monomers

C	7.06470700	0.30835100	0.00001100
H	7.12166800	0.95416800	-0.88446800
H	7.95179600	-0.33455300	0.00000300
H	7.12167000	0.95414700	0.88450500
C	5.77293800	-0.51747600	0.00000100
H	5.76148900	-1.17831700	0.87802900
H	5.76149400	-1.17831200	-0.87803200
C	4.50211400	0.34266300	0.00000300
H	4.51423500	1.00443900	-0.87860700
H	4.51423600	1.00443900	0.87861500
C	3.20356500	-0.47480100	0.00000400
H	3.19239500	-1.13637500	0.87862100
H	3.19239800	-1.13637700	-0.87861200
C	1.93359400	0.38654800	-0.00000100
H	1.94493100	1.04815100	-0.87856400
H	1.94492200	1.04814600	0.87856500
C	0.63488100	-0.43080000	-0.00001000
H	0.62353400	-1.09240300	0.87856400
H	0.62354200	-1.09239800	-0.87858700
C	-0.63488100	0.43080000	-0.00001300
H	-0.62353700	1.09239900	-0.87859000
H	-0.62353900	1.09240200	0.87856100
C	-1.93359400	-0.38654800	-0.00001200
H	-1.94492500	-1.04814900	0.87855200

H	-1.94492800	-1.04814800	-0.87857700
C	-3.20356500	0.47480100	-0.00000900
H	-3.19240100	1.13637300	-0.87862700
H	-3.19239300	1.13637900	0.87860500
C	-4.50211400	-0.34266300	0.00000000
H	-4.51423100	-1.00443800	0.87861100
H	-4.51424100	-1.00444000	-0.87861000
C	-5.77293800	0.51747600	0.00000700
H	-5.76149700	1.17831300	-0.87802400
H	-5.76148600	1.17831600	0.87803600
C	-7.06470700	-0.30835100	0.00001600
H	-7.12166300	-0.95415600	0.88450400
H	-7.95179600	0.33455300	0.00002100
H	-7.12167500	-0.95415900	-0.88446900

(2) The Cartesian coordinates of PTFE with 6 monomers

C	-0.65253800	0.43876600	0.12666400
C	0.65249400	-0.43891900	0.12678500
C	1.97022900	0.39020500	-0.09468700
C	3.27683100	-0.37508100	0.32740000
C	4.59412000	0.24296900	-0.26525800
C	-1.97030300	-0.39047700	-0.09427500
C	-3.27686200	0.37485200	0.32786600
C	-4.59415800	-0.24265200	-0.26535300
C	-5.90228300	0.22415700	0.45558500
C	-7.23338200	-0.03784100	-0.29423500
H	-8.06690500	0.17110600	0.38410400
F	4.52230800	1.59370300	-0.17391100
F	4.67577300	-0.10266600	-1.57242000
F	3.35900500	-0.35978300	1.67970700

F	3.18401000	-1.65983100	-0.09262400
F	1.89312600	1.53162700	0.63071300
F	2.05546600	0.70780100	-1.40829500
F	0.73343300	-1.07784500	1.31824800
F	0.55913200	-1.36026000	-0.86134300
F	-0.55929200	1.35963000	-0.86191800
F	-0.73334600	1.07828100	1.31780800
F	-1.89298100	-1.53170800	0.63139700
F	-2.05579200	-0.70844700	-1.40777200
F	-3.18372900	1.65980200	-0.09147500
F	-3.35941200	0.35887100	1.68014000
F	-4.52257900	-1.59346500	-0.17480700
F	-4.67567000	0.10373400	-1.57234300
F	-5.83182800	1.56156200	0.69533300
F	-5.99424900	-0.41975000	1.65045300
F	-7.29352400	-1.33659900	-0.69422000
F	-7.33237100	0.76246800	-1.38574400
C	5.90227500	-0.22398200	0.45561300
C	7.23349000	0.03795800	-0.29398800
H	8.06682900	-0.17121300	0.38451200
F	5.99428100	0.41978200	1.65053600
F	5.83177400	-1.56141600	0.69509400
F	7.33273600	-0.76221500	-1.38557900
F	7.29379700	1.33676500	-0.69379300

(3) The Cartesian coordinates of PS with 6 monomers

C	-3.55170700	0.68657300	-0.48439700
H	-3.35012300	0.56074600	0.58665200
C	-5.06949700	0.99741800	-0.63516400
H	-5.19942300	2.08650100	-0.67680600

H	-5.43486400	0.61679400	-1.59683900
C	-5.97918000	0.48749600	0.50761400
H	-5.58130000	0.90935500	1.44145400
C	-7.41061300	1.03738200	0.33204800
H	-7.86246600	0.67412300	-0.59792800
H	-8.05532500	0.72351800	1.15911700
H	-7.40586200	2.13291700	0.29759100
C	-6.00026400	-1.02602900	0.68167900
C	-5.66471100	-1.60254100	1.91468700
C	-6.39811000	-1.88063500	-0.35864600
C	-5.71760900	-2.98676000	2.10719000
H	-5.36029300	-0.95895500	2.73728900
C	-6.45199100	-3.26283000	-0.17405900
H	-6.66091000	-1.46624600	-1.32825000
C	-6.11139200	-3.82315900	1.06115900
H	-5.45151100	-3.40808100	3.07286300
H	-6.75606600	-3.90415900	-0.99666900
H	-6.15238000	-4.89913100	1.20429400
C	-3.12515100	-0.60361600	-1.17807800
C	-3.26056300	-0.75873600	-2.56793000
C	-2.55346100	-1.65824800	-0.45397300
C	-2.84418500	-1.92742100	-3.20847300
H	-3.69522100	0.04173400	-3.16173800
C	-2.13321800	-2.83106100	-1.08824800
H	-2.44564200	-1.56733200	0.62362100
C	-2.27693300	-2.97075900	-2.46987800
H	-2.96128300	-2.02264900	-4.28470500
H	-1.69601000	-3.63327400	-0.50060800
H	-1.95269800	-3.88091600	-2.96627000

C	-2.72312500	1.89976000	-0.98765000
H	-3.07110300	2.79736700	-0.45874500
H	-2.95626000	2.06420900	-2.04719400
C	-1.18261700	1.79790700	-0.83620800
H	-0.87952900	0.83115500	-1.25313200
C	-0.50643600	2.87657400	-1.67572700
C	0.21155800	2.53089700	-2.83011800
C	-0.60569600	4.23886900	-1.34707200
C	0.81339700	3.50683700	-3.63049300
H	0.29779100	1.48273400	-3.10694000
C	-0.00664500	5.21860800	-2.14217000
H	-1.15505600	4.54295400	-0.45998900
C	0.70752600	4.85667600	-3.28845700
H	1.36459700	3.21090700	-4.51895900
H	-0.09799600	6.26562400	-1.86602000
H	1.17424400	5.61812000	-3.90666400
C	-0.75815900	1.84818600	0.65155500
H	-1.29853800	1.07048100	1.20460400
H	-1.08785700	2.80379200	1.07847100
C	0.76029800	1.68678400	0.93154900
H	1.28470200	2.37977600	0.26416900
C	1.07759900	2.12045600	2.35854200
C	1.81722700	3.28783000	2.59893500
C	0.62161400	1.39148800	3.46960500
C	2.09699000	3.71555200	3.90060000
H	2.17840700	3.87026900	1.75444100
C	0.89654800	1.81376300	4.77218600
H	0.04720900	0.48104600	3.32029500
C	1.63740000	2.97869300	4.99416700

H	2.67452100	4.62248500	4.05762600
H	0.53293100	1.23157700	5.61464900
H	1.85303700	3.30646600	6.00702900
C	1.24233800	0.24700800	0.62919000
H	0.95177500	-0.02378200	-0.39288100
H	0.70223300	-0.44742000	1.28433800
C	2.76500000	-0.01274300	0.79472900
H	3.06375100	0.40075900	1.76682800
C	3.01956000	-1.51412700	0.85694300
C	3.37547900	-2.11939800	2.07100800
C	2.85087600	-2.33998600	-0.26551100
C	3.56516700	-3.50145600	2.16483200
H	3.50506100	-1.49912600	2.95517700
C	3.03916800	-3.72045700	-0.17899800
H	2.57700400	-1.90399600	-1.22227900
C	3.39831400	-4.30858100	1.03759100
H	3.84435000	-3.94449700	3.11712500
H	2.91053900	-4.33702800	-1.06438600
H	3.54800200	-5.38248500	1.10387600
C	3.58699900	0.72983000	-0.28447300
H	3.25369500	1.77296200	-0.29381500
H	3.34631700	0.33103200	-1.27818300
C	5.12445000	0.73284300	-0.08220100
H	5.51612000	1.65676800	-0.52731700
H	5.34687300	0.79739000	0.98983500
C	5.88883900	-0.43023800	-0.68734900
C	6.56852800	-1.35926700	0.11123600
C	5.96431900	-0.57809700	-2.08205100
C	7.29238400	-2.40992200	-0.46014200

H	6.52369000	-1.26531200	1.19294100
C	6.68425500	-1.62562400	-2.65939700
H	5.45903600	0.14035700	-2.72420000
C	7.35214600	-2.54867500	-1.84837300
H	7.80665100	-3.12039900	0.18120300
H	6.73024000	-1.71763100	-3.74128900
H	7.91465100	-3.36389000	-2.29455000

Reference

[1] A.R. Blythe, T. Blythe, D. Bloor, Electrical properties of polymers, Cambridge University Press, New York, 2005.
